# Source Acquisition Device Identification from Recorded Audio Based on Spatiotemporal Representation Learning with Multi-Attention Mechanisms

**DOI:** 10.3390/e25040626

**Published:** 2023-04-06

**Authors:** Chunyan Zeng, Shixiong Feng, Dongliang Zhu, Zhifeng Wang

**Affiliations:** 1 Hubei Key Laboratory for High-Efficiency Utilization of Solar Energy and Operation Control of Energy Storage System, Hubei University of Technology, Wuhan 430068, China; 2 National Engineering Research Center for Multimedia Software, School of Computer Science, Wuhan University, Wuhan 430072, China; 3 Department of Digital Media Technology, Central China Normal University, Wuhan 430079, China

**Keywords:** audio forensics, spatiotemporal representation learning, attention mechanism, temporal convolution networks

## Abstract

Source acquisition device identification from recorded audio aims to identify the source recording device by analyzing the intrinsic characteristics of audio, which is a challenging problem in audio forensics. In this paper, we propose a spatiotemporal representation learning framework with multi-attention mechanisms to tackle this problem. In the deep feature extraction stage of recording devices, a two-branch network based on residual dense temporal convolution networks (RD-TCNs) and convolutional neural networks (CNNs) is constructed. The spatial probability distribution features of audio signals are employed as inputs to the branch of the CNN for spatial representation learning, and the temporal spectral features of audio signals are fed into the branch of the RD-TCN network for temporal representation learning. This achieves simultaneous learning of long-term and short-term features to obtain an accurate representation of device-related information. In the spatiotemporal feature fusion stage, three attention mechanisms—temporal, spatial, and branch attention mechanisms—are designed to capture spatiotemporal weights and achieve effective deep feature fusion. The proposed framework achieves state-of-the-art performance on the benchmark CCNU_Mobile dataset, reaching an accuracy of 97.6% for the identification of 45 recording devices, with a significant reduction in training time compared to other models.

## 1. Introduction

Source acquisition device identification from recorded audio comprises research on the identification of recording devices by analyzing the intrinsic characteristics of audio [1] and is an important topic in the field of digital audio passive forensics [2,3,4]. In secure forensic applications, recording device identification can be used to authenticate the source of audio files to confirm security, and device-related information in the audio is equivalent to an embedded passive watermark, which is a unique fingerprint belonging to the attributed device [5,6]. In addition, it can also assist in determining whether the audio has been tampered with by detecting whether the device information in the audio is consistent, thereby confirming the integrity and reliability of the data [4,7]. Similarly, it can be used to determine the authenticity of a voice in order to identify whether it is AI-generated or not.

Research on recording device source identification focuses on the identification of the source recording device of the audio data, i.e., the attribution category of the target audio is derived by matching with the database through identification. This identification process relies on the signature information of the recording device embedded in the audio data, which is a kind of recording-device-related discriminatory information that is generated in the process of recording the target sound using the recording device. The target audio is often regarded as a part of the human voice perception system, but ambient information is also recorded during the recording process. Device-related information that is not easily perceptible to the human ear is also embedded during the audio generation process. This device-related information is derived from differences in the transfer functions of different devices during recording, which are due to differences in the selection of recording-related components and the design of electronic circuits within each device, causing convolutional distortion in the input speech during recording, leaving device-signature information in the recording [8]. Existing studies show that this information is mainly distributed in the low- and medium-frequency bands and is not easily detectable by the human ear.

The development of research on audio recording device identification in the existing literature has gone through several stages, and the existing methods can be divided into three main stages. The first class of methods is based on research on feature engineering of audio signals, such as the Mel frequency cepstrum coefficient (MFCC) [8,9] and the band energy difference (BED) [5] method to extract spectral-like features or other distinguishing features using acoustically relevant a priori knowledge, as well as voice activity detection (VAD) [10] and spectral subtraction [11] methods to reduce irrelevant signals in features. The second class of methods is based on machine learning models, such as inputting features into supervised machine learning models such as support vector machine (SVM) [8] and the Gaussian mixture model (GMM) [12,13,14], which distinguish feature data by mapping them to a high-dimensional space; during model training, the information of feature data in each dimension is transformed into probability density information corresponding to each category. The third class of methods is representation learning based on neural network models, applying deep learning algorithms to the recording device recognition process and constructing network models that fit with the feature data, such as using deep neural networks (DNNs) to refine feature data to improve information density [15] and using CNNs for deep feature extraction from the feature matrix [16,17].

With the widespread streaming of media on the Web, research on streaming data has become a hot topic, among which spatiotemporal representation learning has been rapidly developed due to its sensitivity to the spatiotemporal properties of streaming data [18,19]. Spatiotemporal representation learning can automatically learn hierarchical feature representations from spatiotemporal-type data based on the powerful function approximation capability of deep learning; it is particularly effective for streaming data such as video or audio, and some researchers have used it in audio–visual tasks via a two-branch attention mechanism [20] to model the relation between the global feature of one modality and the local features of another. Similarly, we used a dual-attention mechanism for information complementation in audio understanding for features from different abstraction levels. Inspired by spatiotemporal representation learning, we propose a spatiotemporal information fusion network based on RD-TCN and CNN for recording device identification and incorporate multi-attention mechanisms in this paper. The temporal representation part is constructed with a TCN based on residual dense blocks, and the spatial representation part is constructed with a CNN for deep spatial feature extraction. We use a spatial attention mechanism, temporal attention mechanism, and branch attention mechanism according to the characteristics of feature information to automatically learn the weight assignment to multi-dimensional feature parameters and achieve efficient feature fusion, respectively. The main contributions of this paper are summarized as follows:

- In this paper, we propose a two-branch network to implement spatiotemporal representation learning for recording device identification. The extraction of deep temporal features is performed by a residual dense TCN, and the extraction of deep spatial features is performed by a CNN. The whole representation learning process is optimized by a structured loss function. The implementation codes of this research are available at https://github.com/CCNUZFW/STRLMA (accessed on 20 March 2023).
- In order to collaborate with the spatiotemporal representation network to obtain a better representation of recording devices, we design three attention mechanisms: a spatial attention mechanism, temporal attention mechanism, and branch attention mechanism. The spatial and temporal attention mechanisms assign weights to the input features Gaussian super vector (GSV) and MFCC, respectively, to enhance the representativeness of both features. The branch attention mechanism is applied to the fusion of two-way branches to promote the learning of key information in the fusion process.
- Compared with six baseline methods, the proposed framework achieves state-of-the-art performance on the benchmark CCNU_Mobile dataset, reaching an accuracy of 97.6% for the identification of 45 recording devices, with a significant reduction in training time compared to baseline models.

The rest of this paper is organized as follows. Section 2 is a review of related work in the existing literature. In Section 3, we provide a problem definition of this research and summarize the notations that appear in this paper. In Section 4, we introduce the main framework of this paper, describing in detail the spatiotemporal representation learning model for extracting sound source information. Section 5 focuses on the experimental validation phase and develops the analysis of the experimental results. Section 6 presents the conclusion and future research outlook.

## 2. Related Work

In this section, we review related studies on audio recording device identification and classify them into the following two categories based on the technical characteristics of each method.

### 2.1. Recording Device Identification Based on Traditional Feature Engineering and Machine Learning

In the early stage, some researchers drew on the research in fields such as speaker recognition and used features from these areas as features for source device identification, which has achieved good results. Hanilci et al. [8] proposed a device source identification feature based on MFCC and used an SVM classifier and a vector quantization (VQ) classifier to classify MFCC features in the back end. Their results show that the SVM classifier is better than the VQ classifier. Although this method achieves good results, it also has some drawbacks. For example, most of the recording segments are obtained by recording the human voice, which contains too much speaker information. Therefore, Aggarwal et al. [11] proposed, for the first time, extracting MFCC features from noise spectrum signals of non-speech signals. This approach is better than extracting MFCC features from speech segments based on the analysis of experimental results. The features applied in these methods are not specifically designed for recording device identification.

In addition to MFCC using the speaker recognition domain, GSV [21] has been introduced into the device source identification domain. GSV features are mean vectors in GMM [22], and in device source identification involving GSV features, it is common to use MFCC features to train GMM models, transform the MFCC feature recognition problem into a GMM probability distribution problem, and extract the GSV features. Kotropoulos et al. [12] obtained a GMM model by training MFCC features, extracted GSV features from it to characterize different categories of device source information, and used a neural network with a radial-basis function kernel in the back end for classification, which achieved a good recognition accuracy. Although the original GSV features can achieve certain results in the field of device source identification, the GSV features are probability space density functions containing speaker and device sources, which are not necessarily applicable for direct characterization of device source information. Therefore, Jiang et al. [13] proposed a kernel-based GSV feature in order to make GSV features more applicable to device source identification tasks. This feature projects the traditional GSV into another device information space. Comparative experiments are conducted at the back end using SVM and a sparse representation-based classifier (SRC). The experiments prove that the kernel-based GSV feature achieves better results. These models do not scale well for newly registered audio samples or recording devices.

Although traditional acoustic features have achieved good recognition results in device source identification, most of these traditional features are constructed based on the human ear auditory system and cannot fully characterize the variability among recording devices. Jin et al. [23] proposed a device source identification method based on device source noise estimation. This method first used the silent segment speech signal to extract the device source noise signal as a device source carrier; then, the spectral features were extracted on this carrier, and an SVM classifier was used on the back end to conduct experiments on a dataset containing 24 different models of cell phones. The proposed features performed best, with the highest recognition accuracy of 94.53%. Although the features based on the above method achieve high identification accuracy, the computational effort increases exponentially due to the complexity of the mapping function in the feature extraction process. Therefore, in order to simplify the computational process and save time cost, Luo et al. [5] proposed a simpler spectral feature extraction method. This method first divides the device source speech signal into frames and then performs Fourier transform directly and characterizes the device source differences by calculating the BED between two adjacent frames. This method is relatively intuitive and also effectively reduces the computational effort while still achieving good results in experiments on a dataset of 141 device sources. These shallow recognition models have limited ability to handle a large number of speech samples.

### 2.2. Recording Device Identification Based on Deep Learning

In recent years, deep learning has been widely used and achieved improved performances in many fields [24,25], mainly due to the powerful representation learning capability of deep learning [26,27]. Li et al. [15,28] proposed two kinds of deep representation features based on supervised learning: one of the deep learning features based on supervised learning uses MFCC features and labels to train a DNN, then extracts bottleneck features from the middle layer of the DNN. The other deep learning feature uses MFCC features and labels to train the deep autoencoding network, then takes the bottleneck features of the middle layer as the device source features afterwards. The experimental results show that the deep representation learning features outperformed the general features. Combined with previous developments in the field of device source identification, spectral features and 2D image form features can also work well in the field of device source identification.

Influenced by research in image processing and recognition, several researchers have proposed methods to transform audio signals into two-dimensional images to characterize device source features. Qin et al. [16] trained CNN models by transforming speech signals into a spectral map as input. Lin et al. [29] combined deep learning methods with traditional spectral features, using an attention mechanism to assign adaptive network feature weights to different bands of the spectrum in the same device source speech. In response to the methods emerging in the field of traditional device source identification, Qi et al. [30] first used both denoising processing and spectral subtraction to obtain a noisy signal, then extracted the Fourier histogram coefficients of the noisy signal as the input features of a deep network model. Deep learning models can not only train large datasets, but also have powerful generalization and migration properties. Baldini et al. [17] used shallow CNN models in the back end to surpass traditional classification methods and achieved better results. However, the fitting of shallow networks does not fully reflect the fitting effect of deep learning. In a previous study [1], we proposed a multifeature fusion recording device source identification method based on an attention mechanism, using deep representation learning to extract key information features for device source identification. These methods use deep representation learning methods to obtain better results compared to shallow models. Furthermore, these methods focus more on the representation learning of spatial features and less on the representation learning of temporal features. Therefore, in this paper, we propose a recording device identification method based on spatiotemporal representation learning and a multi-attention mechanism, considering the spectral spatial properties of GSV features and the temporal characteristics of MFCC features, and construct a model of the spatiotemporal characteristics of recording-device-related information in audio data.

## 3. Preliminaries

In this section, we first formally define the task of audio recording device identification. Then, we explain the definitions related to deep temporal features and deep spatial features used in this paper. Next, we describe the proposed multi-attention mechanism. Table 1 shows some important notations. The following section provides a more detailed explanation of their roles.

### 3.1. Problem Definition

**Definition 1**  (Audio recording device identification problem). *Audio recording device identification aims at detecting the recording device identity of a test audio signal ($A^{t}$) from an enrollment database ($\left\{A_{d}^{e}|d=1,2,\ldots ,D\right\}$) as:*

(1) $$d^{*}=\arg\max_{d}\left\{f\left(A_{1}^{e},A^{t};\varphi \right),f\left(A_{2}^{e},A^{t};\varphi \right),\ldots ,f\left(A_{D}^{e},A^{t};\varphi \right)\right\},$$

**Figure 1:**
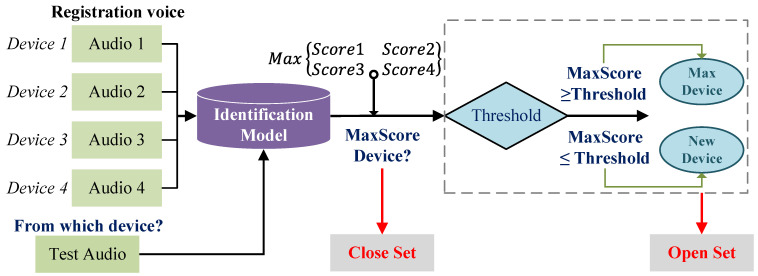
Flow chart of recording device identification.

*where f(·) denotes a function used to calculate the similarity; **φ** stands for the parameters of the back end; $A^{e}$ and $A^{t}$ are the enrollment and test device features, respectively; and D> 1 denotes the number of enrollment devices. If $A^{t}$ can never be outside of the D registered devices, then the recording device identification problem is a closed-set problem; otherwise, it is an open-set problem. A flow chart of the recording device identification process is presented in Figure 1.*

### 3.2. Deep Feature and Multi-Attention Mechanism Definition

**Definition 2**  (Deep temporal feature). *The deep temporal features are extracted from the MFCC by RD-TCN. For time-series feature data, which are generally frame-processed features, it is necessary to not only analyze the information of feature vectors within frames but also to analyze the information association between frames. Deep temporal features are extracted from the original features using deep learning, which requires the use of networks with sequence modeling capabilities, such as RNN and LSTM; a modified TCN is used in this paper. The extracted deep temporal features reflect the temporal information in the feature data, including the analysis of similarity information and mutation information between adjacent frames, as well as the analysis of long-term fluctuation information.*

**Definition 3**  (Deep spatial feature). *The deep spatial features are extracted from the GSV by CNN. GSV is a probability distribution feature extracted by GMM that maps the original feature data to the high-dimensional feature space during GSV extraction, and the extracted GSV reflects the statistical information of Gaussian distribution in the sample space. The extracted deep spatial features reflect the spatial information in the feature data, including the analysis of the correlation between different Gaussian components in the GSV feature matrix, as well as the analysis of the local associations of different regions in the two-dimensional space of the feature matrix.*

**Definition 4**  (Multi-Attention mechanism). *The multi-attention mechanism proposed in this paper consists of three attention mechanisms, namely a spatial attention mechanism, temporal attention mechanism, and branch attention mechanism, which act in different stages for feature reconstruction and feature fusion, respectively. The core idea is learning the importance distribution in the input features according to the corresponding attention blocks, then enhancing the information related to classification in the features by weight assignment. The reconstruction of the original features by the spatial attention mechanism and the temporal attention mechanism highlights the classification-related information and improves the utilization of effective information in the model. The fusion of the two-way network information by the branch attention mechanism achieves complementary utilization of spatiotemporal feature information and improves the information density.*

## 4. Methods

**Figure 2:**
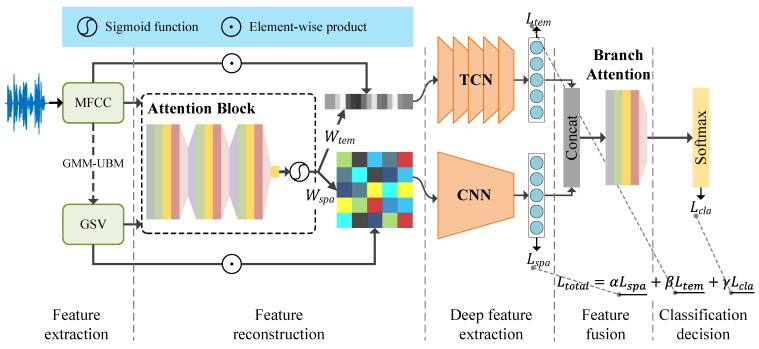
Spatiotemporal representation learning framework ($W_{tem}$, $W_{spa}$: weights of temporal and spatial features; $L_{total}$: overall loss of the model; $L_{spa}$: spatial feature loss; $L_{tem}$: temporal feature loss; $L_{cla}$: classification loss; α,β,γ: proportion coefficients of the three losses).

The spatiotemporal representation learning framework includes feature reconstruction, deep feature extraction, feature fusion, and classification decisions, and the whole task is jointly trained with end-to-end architecture. The overall framework is shown in Figure 2.

- The feature reconstruction phase is divided into temporal feature reconstruction and spatial feature reconstruction. Temporal feature reconstruction assigns adaptive weights to the features at the temporal scale through the attention mechanism and marks the significant sequences to improve the effects of important feature sequences on the model. Similarly, reconstruction for spatial information involves learning the spatial feature information using the attention mechanism to assign different weights to enhance spatial features.
- In the deep feature extraction stage, the temporal branch based on RD-TCN is used to extract the deep temporal features, and the spatial branch based on CNN is used to extract the deep spatial features.
- In the feature fusion phase, a branch attention mechanism is designed for the fusion of deep temporal features and deep spatial features.
- In the classification decision phase, we apply a multiloss joint computation strategy in order to build an end-to-end network system and optimize the learning process of the two-way branch network and the decision end.

The process of the proposed spatiotemporal representation learning model is shown in Algorithm 1.

### 4.1. Feature Reconstruction Phase

#### 4.1.1. Feature Reconstruction of MFCC Based on a Temporal Attention Mechanism

The MFCC feature is a frequency cepstrum feature based on short-term Fourier transform, which is one of the most commonly used features in the field of source recording device identification. The extraction process of MFCC includes preprocessing (frame splitting and windowing), fast Fourier transform (FFT), Mel filtering, logarithmic operation, and discrete cosine transform (DCT). The specific extraction process is as follows:

- First, in order to obtain a stable representation of the audio signal in the frequency domain, the audio signal (A) needs to be framed. A Hamming window with frame length (fl) and frame shift (fs) is used to obtain the short time frames;
- Then, the frequency spectral information of each frame is obtained by performing a fast Fourier transform on the framed and windowed signal;
- Then, Mel-scale triangular filters are used to filter the frequency spectra of frames;
- Then, the logarithmic amplitude spectrum at the output of each filter bank is calculated, and the *M*-dimension MFCC vectors are obtained by DCT calculation.

Although MFCC is capable of representing time-based changes in audio information, this raw information contains a large amount of redundant information. The temporal attention mechanism is a typical technique used for time-series data classification to attenuate and remove the noisy or irrelevant parts. In the process of device source identification, the temporal attention mechanism possesses strong practical significance. For example, the presence of many human voices in the device source speech interferes with the device source information, and sequence segments with many similar human voices can be attenuated by the temporal attention mechanism, which can be used to overcome the problem of masking and irrelevant signals. Specifically, the attention mechanism can be used to measure the relevance of each time step, and the temporal attention weights provide significance to meaningful values in the sequence, which helps to distinguish similar regions and mark important information. The temporal attention mechanism constructs weights in the same way as the spatial attention mechanism, using convolutional layers to construct learnable parameters, then adjusting the temporal feature maps.

The temporal attention mechanism proposed in this paper first extracts the internal correlation between the contextual relationships and feature vectors in the temporal feature spectrum through a convolutional layer, then assigns weights to the time series using a single-scale maximum pooling layer to control the importance coefficients of each feature vector on the time scale. Finally, weights indicating the degree of importance are assigned to each time node in the temporal feature spectrum by multiplying by the input feature sequence.

**Figure 3:**
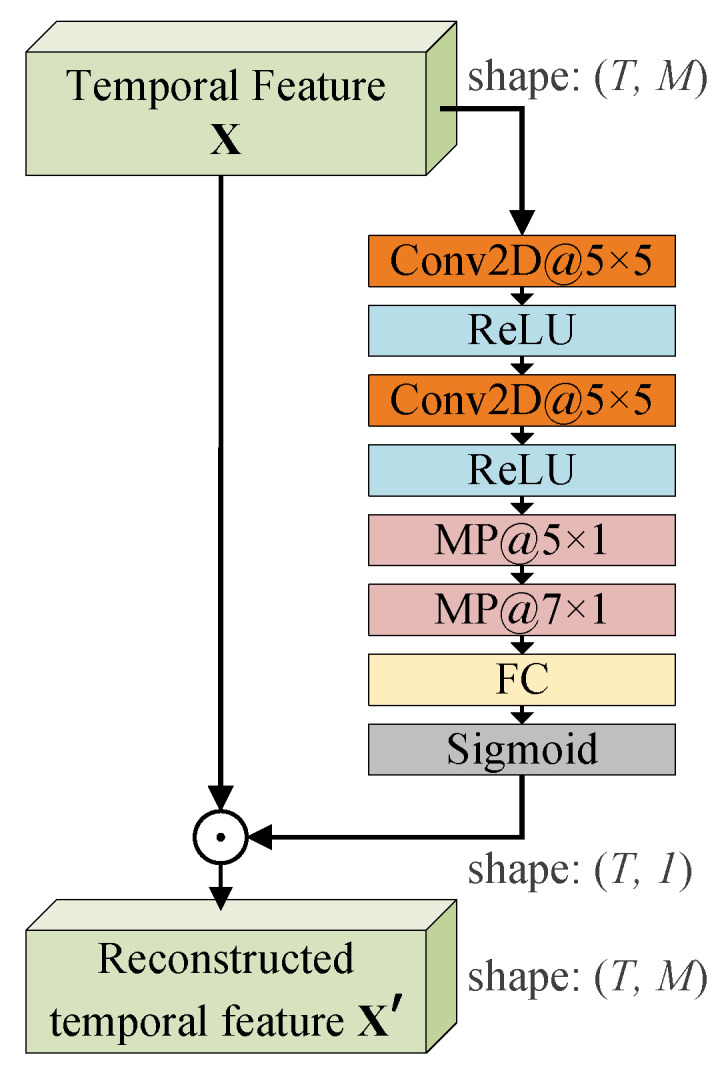
Temporal attention mechanism structure diagram. (*T*: number of MFCC vectors; *M*: length of per MFCC vector; ⊙: element-wise product (Hadamard product of features-by-column and weight column vector ($W_{tem}$))).

In summary, the temporal attention mechanism with X as input and $X^{\prime }$ as output can be formulated as

(2) $$\left\{\begin{array}{c} W_{tem}=F_{tem}\left(X,\theta_{tem}\right)=\sigma \left(FC\left(MP\left(\delta \left({Conv}_{2}\left(\delta \left({Conv}_{1}\left(X\right)\right)\right)\right)\right)\right)\right),\\ X^{\prime }=W_{tem}X. \end{array}\right.$$

where σ is sigmoid activation; δ is ReLU activation; and FC, MP, and Conv denote the fully connected layer, maximum pooling layer, and convolutional layer, respectively. In the training phase, attentional temporal pooling and temporal convolutional networks are jointly trained to guide our model for effective information extraction in the temporal dimension. The temporal attention mechanism is designed to emphasize the importance distribution of sequential information, and its network structure and parameters are designed to learn the temporal correlation of input features on the time scale. The specific structure is shown in Figure 3.
**Algorithm 1** The proposed spatiotemporal representation learning model.
  **Input**: MFCC feature X: a sequence of MFCC vectors $\left\{x_{1},x_{2},\ldots ,x_{T}\right\}$, GSV feature       G: a feature matrix of shape (M,K).
  **Output**: The prediction of the attributed recording device for the input sample
 **1** Reconstruct the input temporal feature X into $X^{\prime }$ by temporal attention mechnism:$$\left\{\begin{array}{c} W_{tem}=F_{tem}\left(X,\theta_{tem}\right)=\sigma \left(FC\left(MP\left(\delta \left({Conv}_{2}\left(\delta \left({Conv}_{1}\left(X\right)\right)\right)\right)\right)\right)\right),\\ X^{\prime }=W_{tem}X. \end{array}\right.$$
 **2** Reconstruct the input spatial feature G into $G^{\prime }$ by spatial attention mechnism:$$\left\{\begin{array}{c} W_{spa}=F_{spa}\left(G,\theta_{spa}\right)=\sigma \left(FC\left(MP\left(\delta \left(Conv\left(G\right)\right)\right)\right)\right),\\ G^{\prime }=W_{spa}G. \end{array}\right.$$
 **3** Extract deep spatial features $\hat{G}$ through CNN blocks of spatial network branch:$$\hat{G}=F_{CNN}\left(G^{\prime }\right).$$
 **4** Extract deep temporal features $\hat{X}$ through RD-TCN blocks of the temporal network branch:$$\hat{X}=F_{RD-TCN}\left(X^{\prime }\right).$$
 **5** Compute spatial feature loss $L_{spa}$:$$\left\{\begin{array}{c} {\hat{y}}_{spa}^{\left(d\right)}=\arg\max_{d=d^{*}}Softmax(W_{CNN}\hat{G}+b),\\ L_{spa}=-\sum_{d=1}^{D}y_{d}\log\left({\hat{y}}_{spa}^{\left(d\right)}\right). \end{array}\right.$$
 **6** Compute temporal feature loss $L_{tem}$:$$\left\{\begin{array}{c} {\hat{y}}_{tem}^{\left(d\right)}=\arg\max_{d=d^{*}}Softmax(W_{RD-TCN}\hat{X}+b),\\ L_{tem}=-\sum_{d=1}^{D}y_{d}\log\left({\hat{y}}_{tem}^{\left(d\right)}\right). \end{array}\right.$$
 **7** Concatenate deep temporal features $\hat{X}$ and deep spatial features $\hat{G}$, and assign weights by branch attention mechanism to achieve feature fusion: $$\left\{\begin{array}{c} Y=[\hat{X},\hat{G}],\\ W_{bra}=F_{bra}\left(Y,\theta_{bra}\right)=\sigma \left(FC\left(MP\left(\delta \left({Conv}_{2}\left(\delta \left({Conv}_{1}\left(Y\right)\right)\right)\right)\right)\right)\right),\\ Y^{\prime }=W_{bra}Y. \end{array}\right.$$
 **8** Compute classification loss $L_{cla}$:$$\left\{\begin{array}{c} {\hat{y}}_{cla}^{\left(d\right)}=\arg\max_{d=d^{*}}Softmax(W_{cla}Y^{\prime }+b),\\ L_{cla}=-\sum_{d=1}^{D}y_{d}\log\left({\hat{y}}_{cla}^{\left(d\right)}\right). \end{array}\right.$$
 **9** Compute the overall loss $L_{total}$:$$L_{total}\;=\;\alpha L_{spa}+\beta L_{tem}+\gamma L_{cla}.$$
**10** Predict the source recording device $d^{*}$.


#### 4.1.2. Feature Reconstruction of GSV Based on the Spatial Attention Mechanism

GSV features have been shown to be effective in research on recording device source identification [12,13]; the core idea is that the probability distribution of any shape can be approximated by multiple Gaussian distribution functions. GSV is constructed through the adoption of GMM. In adapting the parameters of GMM by the MAP adaptation algorithm using the target device data, the target device GMM parameters are obtained, and the mapping of feature data in the GMM feature space in the parameter learning process is highly correlated with the target category in the mapping direction.

The discrepancy information in the GMM model of each target device source mainly exists in its mean value, and the mean vector thus extracted is the GSV feature. For the number of Gaussian components (*K*), the dimension of each MFCC vector is *M*, and the mean value of each target model is a matrix of (M,K). The GSV features are obtained by concatenating the mean vectors of each target model.

The extraction process of GSV features involves the following three steps:

***Step 1***: If a audio data correspond to a feature (X), where $X=\{x_{1},x_{2},\ldots x_{T}\}$, and assuming its dimensionality is *M*, the formula used to calculate its likelihood function is:

(3) $$p\left(x_{t}\left|\lambda \right.\right)=\sum_{k=1}^{K}\omega_{k}p_{k}\left(x_{t}\right),$$

where this density function is obtained by weighting *K* single Gaussian density functions ($p_{k}\left(x_{t}\right)$), where the mean $\mu_{k}$ and the covariance $\Sigma_{k}$ of each Gaussian component are of sizes (1,M) and (M,M), respectively.

(4) $$p_{k}\left(x_{t}\right)=\frac{1}{{\left(2\pi \right)}^{M/2}{\left|\Sigma_{k}\right|}^{1/2}}\exp\left\{-\frac{1}{2}{\left(x_{t}-\mu_{k}\right)}^{T}\Sigma_{k}^{-1}\left(x_{t}-\mu_{k}\right)\right\},$$

where the mixture weights ($w_{k}$) satisfy $\sum_{k=1}^{K}w_{k}=1$. Assuming that λ denotes the set of model parameters, $\lambda =\{w_{k},\mu_{k},\Sigma_{k}|k=1,2,\ldots ,K\}$, which is derived by expectation maximization (EM) iterative training.

***Step 2***: Using the EM algorithm to estimate the iterative parameter (λ), first, assign λ an initial value; then, estimate the new parameter ($\lambda^{\prime }$) in order to satisfy $p\left(X\left|\lambda^{\prime }\right.\right)\geq p\left(X\left|\lambda \right.\right)$. In order to ensure that the likelihood of λ under $\lambda^{\prime }$ is the highest possible, the new parameters are iteratively trained again; the estimation formulae of each parameter are shown in Equations (5)–(7).

(5) $$w_{k}=\frac{1}{T}\sum_{t=1}^{T}p\left(k|x_{t},\lambda \right),$$

(6) $$\mu_{k}=\frac{\sum_{t=1}^{T}p\left(k|x_{t},\lambda \right)x_{t}}{\sum_{t=1}^{T}p\left(k|x_{t},\lambda \right)},$$

(7) $$\Sigma_{k}=\frac{\sum_{t=1}^{T}{p\left(k|x_{t},\lambda \right)\left(x_{t}-\mu_{t})\left(x_{t}-\mu_{t}\right)\right.}^{T}}{\sum_{t=1}^{T}p\left(k|x_{t},\lambda \right)}.$$

where $w_{k}$ denotes the mixture weights, $\mu_{k}$ denotes the mean, and $\Sigma_{k}$ is the covariance matrix.

***Step 3***: Finally, the feature vectors of *D* devices are adapted by MAP to obtain the device-specific source GMM, and the mean vector of GMM is extracted, which is the GSV features.

In device source identification, the information density of different parts of the spatial features is not equal, and only the parts relevant to the classification task need to be attended to. The spatial attention mechanism finds the most important parts of the feature map for processing and displays the importance in the form of probability maps or probability feature vectors to emphasize important information and suppress useless information. The spatial attention mechanism designed in this paper aims to reconstruct the features by including weights for the device source features so that the features retain sufficient device-related spatial information. The spatial attention mechanism uses convolution and pooling to construct learnable parameters, which are jointly optimized with the whole model to construct efficient device source features.

**Figure 4:**
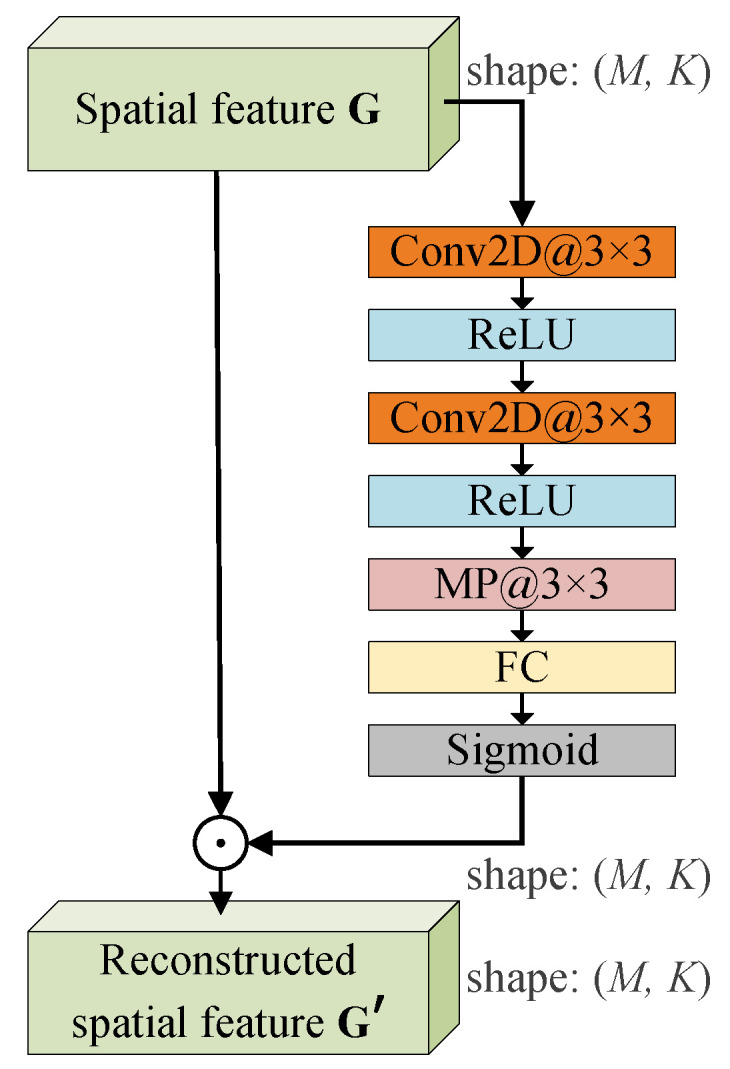
Spatial attention mechanism structure diagram. (*M*: length of per MFCC vector; *K*: number of Gaussian mixture components; ⊙: element-wise product (Hadamard product of spatial feature and weight matrix ($W_{spa}$))).

The spatial attention mechanism proposed in this paper captures the spatial relationships in the input feature maps through a 2D convolutional layer and controls the weight assignment of the importance of spatial information by maximum pooling. Then, spatial-attention-based weight assignment is achieved by multiplying the corresponding elements in the input features. In summary, the spatial attention mechanism, with G as the input and $G^{\prime }$ as the output, can be formulated as:

(8) $$\left\{\begin{array}{c} W_{spa}=F_{spa}\left(G,\theta_{spa}\right)=\sigma \left(FC\left(MP\left(\delta \left(Conv\left(G\right)\right)\right)\right)\right),\\ G^{\prime }=W_{spa}G. \end{array}\right.$$

where σ is the sigmoid activation; δ is the ReLU activation; and FC, MP, and Conv denote the fully connected layer, maximum pooling layer, and convolution layer, respectively. The spatial attention mechanism aims to establish a mapping of the importance level of location information, and its network structure and parameters are designed to learn the spatial correlation of the input feature matrix. The specific structure is shown in Figure 4. The structure diagrams of the attention blocks are drawn similarly, but they have different types of inputs and outputs (the temporal dimension in the temporal feature (X) is much larger than the dimension of the spatial feature (G), *T* ≫K>M). They also differ in terms of their structure and parameters set by the design purpose.

### 4.2. Deep Feature Extraction Phase

#### 4.2.1. Deep Spatial Feature Extraction Based on CNN

In this paper, a CNN is used to extract spatial feature information from the input data, and after completing the spatial feature extraction, a fully connected layer is used to remove the location information and reduce the sensitivity of parameters in the subsequent fusion process. CNN networks have excellent processing ability for 2D data. The spatial information in the input data is extracted by transforming the input 2D data into a feature map through feature extraction with a convolutional kernel. The CNN local perception and parameter-sharing feature greatly reduces the network parameters, ensures the sparsity of the network, and preserves the local correlation of the samples. In summary, the CNN block, with $G^{\prime }$ as the input feature and $\hat{G}$ as the output feature, can be formulated as:

(9) $$\hat{G}=F_{CNN}\left(G^{\prime }\right).$$

The CNN used for deep spatial feature extraction is composed of a convolutional layer, pooling layer, and fully connected layer. The convolutional layer is obtained by convolving the feature surface with the local region of the layer’s feature surface through a convolutional kernel; this operation is able to extract the deep features from the feature surface. Setting the convolution kernel as (H,L,C), where *C* is the number of channels and (H,L) is the size of a single convolution kernel, the feature is passed through the convolution calculation to the convolution layer to form the data as:

(10) $$v\left(i,j\right)=f\left(\sum_{h=1}^{H}\sum_{l=1}^{L}\left.\sum_{c=1}^{C^{{}^{\prime }}}a_{h,l,c}^{j}w_{h,l,c}^{i}+b^{i}\right)\right.,$$

where *i* denotes the *i*-th channel of the convolution layer; $C^{\prime }$ denotes the $C^{\prime }$ channels associated with the convolution layer in the *C* channels of the input layer (or pooling layer) ($C^{\prime }\leq C$); $w_{h,l,c}^{i}$ denotes the convolution kernel required for the *i*-th channel of the convolution layer; $a_{h,l,c}^{j}$ is the *j*-th input of the input layer (or pooling layer) (depends on the value of the input layer or pooling layer and the step size); f(·) is the activation function, usually chosen as a ReLU, sigmoid, or tanh function; and $v\left(i,j\right)$ denotes the specific value of the *j*-th value of the *i*-th channel.

The resolution of the eigenfaces is then reduced by pooling operations, while also maintaining the spatial invariance of the eigenfaces.

(11) $$v_{j}^{l}=down\left(v_{j}^{l-1}\right),$$

where $down(v_{j}^{l-1})$ denotes the *j*-th feature mapping after pooling for the l−1-th layer.

After convolution, the data from the previous layer are normalized with different weights by discarding the location information through the fully connected layer to obtain an output result with the following formula for the feature information output from the fully connected layer.

(12) $${\hat{g}}_{j}=f\left(w_{j}^{l}fc\left(v_{j}^{l}\right)+b_{j}\right),$$

where $fc(v_{j}^{l})$ denotes the expansion of the pooling layer into a fully connected form, and ${\hat{g}}_{j}$ is the the *j*-th value of spatial feature information extracted by the CNN.

The spatial feature losses ($L_{spa}$) are expressed as:

(13) $$\left\{\begin{array}{c} {\hat{y}}_{spa}^{\left(d\right)}=\arg\max_{d=d^{*}}Softmax(W_{CNN}\hat{G}+b),\\ L_{spa}=-\sum_{d=1}^{D}y_{d}\log\left({\hat{y}}_{spa}^{\left(d\right)}\right). \end{array}\right.$$

#### 4.2.2. Deep Temporal Feature Extraction Based on RD-TCN

Time-series features are commonly modeled using recurrent neural networks (RNNs) [31,32] and their variants, as they have a recurrent regression structure suitable for modeling time series; however, their performance is still affected by their inability to be parallel, and they have disadvantages such as limited ability to analyze information within a sequence and high training time consumption. In general, CNNs are limited by the convolutional kernel size and cannot capture long-term dependent information well and are therefore considered unsuitable for modeling of time-series information. However, some recent works have shown that specific convolutional neural network structures can also achieve good results; for example, TCN was compared with a variety of RNN structures and found to be capable of matching or even surpassing RNN models in a variety of tasks.

TCN has the advantage of parallel processing of data. Unlike RNN-type networks, which require sequential processing of data in a sequential manner, TCN can perform parallel processing of given temporal data, thereby greatly reducing the training time. In addition, TCN can set parameters such as the number of network layers, convolutional kernel size, and the dilation rate according to the task, thereby indirectly setting the receptive field size to accommodate feature data of different complexities.

TCNs use causal convolution in order to enable the extraction of temporal information. Causal convolution has a unidirectional structure, which means that there is a preceding cause before there is a subsequent effect, and it is a strictly time-constrained model. Pure causal convolution still suffers from the problem that the modeling length of the time scale is limited by the size of the convolutional kernel, and a very large number of hidden layers needs to be stacked in order to extract longer time series of dependencies. Thus, dilation convolution is used to complete the temporal convolution operation. Dilation convolution allows the input to be sampled at intervals during convolution. Therefore, the dilation convolution makes the size of the effective receptive field grow exponentially with the number of layers, allowing the convolution kernel to parse the extracted features. This allows the convolutional network to obtain a sufficiently large receptive field with relatively few layers.

**Figure 5:**
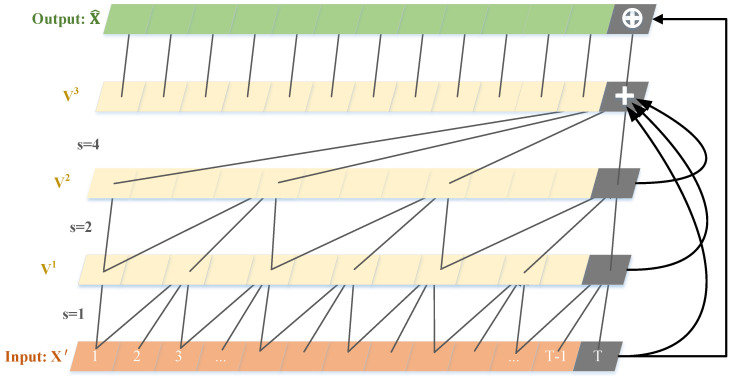
Schematic diagram of a TCN based on residual dense blocks ($V^{l}$: activation values in the *l*-th layer; *s*: dilation rate; +: concatenation operation; ⊕: addition operation).

Here, we introduce improvements on the basis of a TCN by constructing an RD-TCN using residual dense blocks, which further enhances the information utilization compared with a normal TCN. A structural diagram of RD-TCN block is shown in Figure 5. In summary, the RD-TCN block, with $X^{\prime }$ as the input feature and $\hat{X}$ as the output feature, can be formulated as:

(14) $$\hat{X}=F_{RD-TCN}\left(X^{\prime }\right).$$

**Figure 6:**
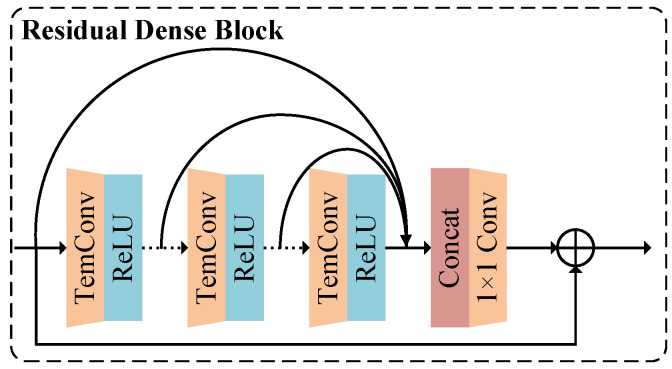
Structure of the residual dense block of the temporal convolution. ⊕: addition operation.

We propose the use of a residual dense structure [33] instead of a residual structure as in an ordinary TCN in order to make efficient use of all the layered information in the convolutional layers through the residual dense blocks. We construct residual dense blocks in TCN to achieve skip-layer connectivity; the residual dense blocks allow the network to transfer information in a cross-layer manner, fusing lower-layer features with higher-layer features to enhance information utilization. The network structure of the residual dense block is shown in Figure 6. In this paper, residual dense blocks are used as building blocks for the RD-TCN, as they contain ordinary residual concatenation layers and dense feature fusion with local residual learning. The residual dense block supports continuous memory, and after extracting multiple layers of local dense features, it further fuses the global features, then adaptively retains the layered features in a global manner, thereby achieving implicit deep supervision.

As shown in Figure 5, the activation values in the *l*-th layer are represented by $V^{l}\in R^{C\times T}$. Each layer has the same number of filters (*C*), which enables us to combine activation values from different layers using skip connections later. When the convolution kernel size is set to three, i.e., the number of time steps beyond which the convolution is applied, the temporal convolution is calculated as

(15) $$V_{t}^{l}=\delta \left(W^{\left(1\right)}V_{t-2s}^{l-1}+W^{\left(2\right)}V_{t-s}^{l-1}+W^{\left(3\right)}V_{t}^{l-1}+b\right),$$

where $V_{t}^{l}$ is the result of the dilated convolution at time *t* of the *l*-th layer, the input to the first layer is the deep temporal feature $X^{\prime }$, and *s* denotes the rate parameter of the dilation convolution. The result obtained after adding the residual dense connections is calculated as

(16) $$\hat{X}=FC\left(\delta \left(W\left[V^{3},V^{2},V^{1},X^{\prime }\right]\right)+X^{\prime }\right),$$

where *W* denotes the a set of weights, where the bias term is omitted for simplicity, and $\left[V^{3},V^{2},V^{1},X^{\prime }\right]$ refers to the concatenation of the feature maps in the axis of the channel.

The temporal feature loss ($L_{tem}$) are expressed as

(17) $$\left\{\begin{array}{c} {\hat{y}}_{tem}^{\left(d\right)}=\arg\max_{d=d^{*}}Softmax(W_{RD-TCN}\hat{X}+b),\\ L_{tem}=-\sum_{d=1}^{D}y_{d}\log\left({\hat{y}}_{tem}^{\left(d\right)}\right). \end{array}\right.$$

### 4.3. Feature Fusion Based on the Branch Attention Mechanism

In this model, attention factors are added to the temporal features to adjust the weights of temporal scales to enhance the temporal feature vectors, and attention factors are also added to the spatial features to adjust the information distribution of spatial features. After that, the temporal and spatial information is extracted, and finally, the information is fused using the branch attention mechanism. This allows for an increase in the efficiency of temporal and spatial information extraction and improves the identification accuracy.

**Figure 7:**
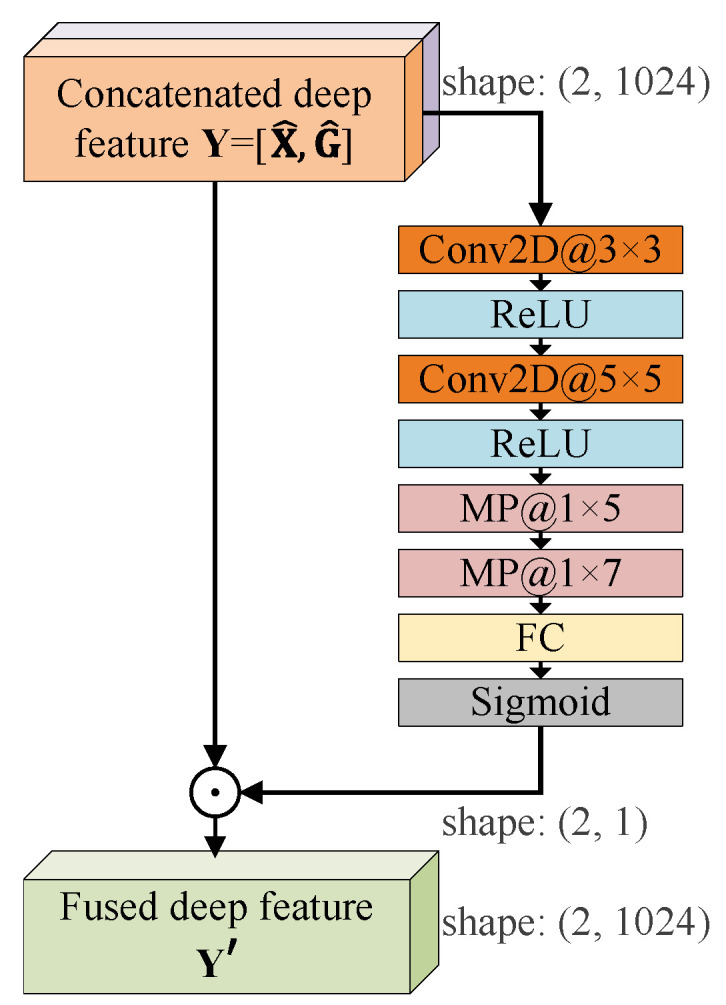
Branch attention mechanism structure diagram. (⊙: element-wise product (Hadamard product of features by column and weight column vector $W_{bra}$)).

This module addresses the problem of fusing different types of features by assigning weights to different types of features through network learning, thereby improving the effects of important features on model training. The attention fusion mechanism designed in this paper includes a weight learning layer and a dot product assignment layer, where the weights to be learned are calculated by the operations of convolution and pooling. In summary, the branch attention mechanism, with $\hat{X}$ and $\hat{G}$ as the input and $Y^{\prime }$ as the output, can be formulated as:

(18) $$\left\{\begin{array}{c} Y=[\hat{X},\hat{G}],\\ W_{bra}=F_{bra}\left(Y,\theta_{bra}\right)=\sigma \left(FC\left(MP\left(\delta \left({Conv}_{2}\left(\delta \left({Conv}_{1}\left(Y\right)\right)\right)\right)\right)\right)\right),\\ Y^{\prime }=W_{bra}Y. \end{array}\right.$$

where $\left[\hat{X},\hat{G}\right]$ refers to the concatenation of the features $\hat{X}$ and $\hat{G}$ in the axis of the channel. The branch attention mechanism fuses the feature outputs of the two-way branches, and its structure and parameters are designed to aid in the feature assignment during the fusion process. The specific structure is shown in Figure 7.

Branch attention can be considered a dynamic branch selection mechanism to determine which branches need to be focused on when used in networks with multibranch structures. The branch attention mechanism first constructs the learnable parameters by convolution, while a set of feature maps can be obtained after convolution, followed by compression of the features into a set of real numbers using a pooling layer, which is also equivalent to a convolution operation with a global sense field. The last layer is the Softmax layer, which adds nonlinearity to the weights. After the Softmax layer, a set of weights is generated for the features, which is used to represent the correlation and importance between the branches of the feature network. The weights are then assigned to each branch, and the features are fused in a dot-product manner.

The classification losses ($L_{cla}$) are expressed as

(19) $$\left\{\begin{array}{c} {\hat{y}}_{cla}^{\left(d\right)}=\arg\max_{d=d^{*}}Softmax(W_{cla}Y^{\prime }+b),\\ L_{cla}=-\sum_{d=1}^{D}y_{d}\log\left({\hat{y}}_{cla}^{\left(d\right)}\right). \end{array}\right.$$

### 4.4. Classification Decision Based on the Joint Loss Function

The overall loss function of the framework proposed in this paper consists of temporal feature loss, spatial feature loss, and classification loss, as shown in the following equation.

(20) $$L_{total}\;=\;\alpha L_{spa}+\beta L_{tem}+\gamma L_{cla},$$

where $L_{total}$ is the overall loss of the model; $L_{tem}$ and $L_{spa}$ are the temporal feature loss and spatial feature loss, respectively; $L_{cla}$ is the classification loss of the decision layer; and α,β,γ denote the proportion coefficients of the three losses to adjust the proportion of each partial loss in the overall loss. The temporal feature loss and spatial feature loss are used to ensure the accuracy of the learning direction of important information in the feature information extraction stage and to update the weights of the branching network over time, respectively.

The classification loss in the decision layer is used to ensure the consistency of the content between the predicted and true categories and to backpropagate the learning update of the model weights. The learning algorithm of the objective function for the loss and network weights is shown in Algorithm 2.  
**Algorithm 2:** Algorithm for model objective function learning
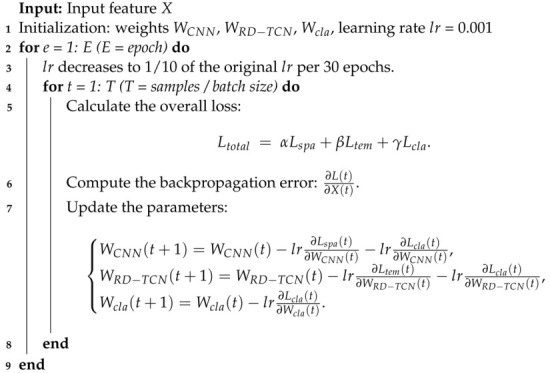


## 5. Experimental Results and Analysis

In this section, we show the extensive experiments conducted to validate the effectiveness and advantages of our method, and we compare the performance of our method with several baselines on a dataset containing 28,890 audio data samples. To validate the effectiveness of each independent part of the framework, we conducted an ablation study based on the idea of the controlled variable method to observe the core modules and key hyperparameters of our method. To obtain the optimal parameters and structure, we conducted experiments using our method for the parameter and structure settings.

### 5.1. Dataset

In this paper, we use the CCNU_Mobile dataset [1] to validate the proposed method. The CCNU_Mobile dataset consists of audio data recorded by 45 different device models and recording devices from 9 different brands, including Apple, Huawei, Honor, Nubia, Oppo, Vivo, Xiaomi, Samsung, and ZTE; the device models are shown in Table 2.

The recording corpus of this dataset is from the TIMIT dataset. During the recording process of the CCNU_Mobile dataset, all the training data in the TIMIT dataset were first spliced into a long audio file with a duration of about 110 min, then played in a quiet dedicated recording studio environment, using 45 devices to record. After the recording was completed, the recorded long audio was cut into segments, with 642 audio samples recorded per device; each recording sample is about 10 s in duration, and the audio samples are all single-channel audio files saved in .wav format with a sampling rate of 32,000 Hz and a bit rate of 512 kbps. We randomly selected 514 samples from each device category for the training set and another 128 samples for the test. The training and test sets accounted for 80% and 20% of the total samples, respectively, and the validation set was 20% of the training set.

### 5.2. Evaluation Metrics

For evaluation purposes, we used classification accuracy (Acc) as the performance metric, which is defined as:

(21) $$Acc\;=\;\frac{S_{cr}}{S_{t}}\times 100\%,$$

where $S_{t}$ denotes the total number of samples participating in the test, and $S_{cr}$ denotes the number of samples that were correctly identified. The recognition results of the samples during the test are computed by the final layer of the model, using the Softmax layer, which maps the output of each neuron in the penultimate fully connected layer to the (0,1) interval to obtain the scores of each category in the multiclassification task and calculates the probability of belonging to each category to obtain the recognition results. For a total of n categories ($Softmax_{d}$) represented by numerical values, where *D* denotes the number of categories and d∈(0,D], the Softmax calculation formula is:

(22) $$P\left(Softmax_{i}\right)=\frac{e^{v_{i}}}{\sum_{d}^{D}e^{v_{d}}},$$

where $Softmax_{i}$ denotes the *i*-th output, and $v_{i}$ denotes the value of the *i*-th category. The final Softmax value obtained for each category sums to 1.

### 5.3. Baselines

In order to evaluate the performance of the methods proposed in this paper, we needed other baselines for comparison. The baseline methods used in the comparison experiments are described below. The details of the baselines are as follows: the Gaussian mixture model–universal background model (GMM-UBM) and MFCC-SVM are two classical methods in the field of recording device source identification, and we used them as reference standards. I-vector and BED features are improved in terms of features to solve the feature information representation problem, and we also used them as features for the baseline methods. In addition, GSV-CNN was added to the baseline method as a representative method of deep learning methods, and a multifeature fusion method using multiple features and deep learning models was added to the baseline method as a novelty.

**GMM-UBM** [14]: This method uses training GMM and calculates probability scores for each category for classification; using UBM to train GMM reduces the computational effort.

**MFCC-SVM** [8]: This method uses MFCC, an inverse spectral feature widely used in the audio recognition field, as a feature and SVM as a classification model.

**I-vector-SVM** [34]: The i-vector method reduces the dimensionality by obtaining the audio feature vector of the high-dimensional target device source, projecting it in the subspace, using factor analysis to eliminate the factors that add redundancy to obtain the low-dimensional feature vector.

**BED-SVM** [5]: This method uses a spectral feature extraction method that calculates the baseband energy difference, which is more intuitive to characterize the device source differences and effectively reduces the computational effort.

**X-vector TDNN-based systems** [35,36]: This method is a high-performance method in the field of speaker recognition. We conducted two experiments on X-vector TDNN-based systems: one using SVM as the back-end classification after extracting X-vectors based on TDNN and the other using a Softmax layer as the back-end direct classification of TDNN.

**GSV-CNN** [17]: This method constructs a representative CNN model for identification, and in this experiment, we used the GSV feature as the input.

**Multifeature fusion** [1]: This method uses three feature inputs and deep and shallow feature fusion using a CNN/DNN.

### 5.4. Experimental Settings

**(1) Framework settings:** To extract the temporal MFCC features, a Hamming window function is chosen; the length of each signal frame is 30 ms, the overlap is 15 ms, and the length of the extracted single MFCC vector is 39. The spatial GSV features are extracted using 64 fitted Gaussian components, and the feature matrix shape is (39, 64). Table 3 and Table 4 show the detailed parameters specific to the three attention mechanisms and the network framework, respectively.

**(2) Training settings:** The method was trained in the same environment as all baseline methods during the training process, with a total of 200 training epochs, a batch size of 128, an initial lr = 0.001, and the lr representing 1/10 of the previous 30 epochs. The experiment was conducted with an Adam optimizer with $\beta_{1}$ = 0.9 and $\beta_{2}$ = 0.999. The experiment-related feature extraction steps and model implementation steps were performed in Matlab 2020a and TensorFlow 2.1. The relevant experimental hardware configuration is as follows: CPU: Intel^®^ Xeon Gold 5218 × 2; GPU: NVIDIA^®^ TITAN RTX (24 GB video memory); and memory: 32 GB.

### 5.5. Results and Discussion

#### 5.5.1. Comparison with Baseline Methods

**Figure 8:**
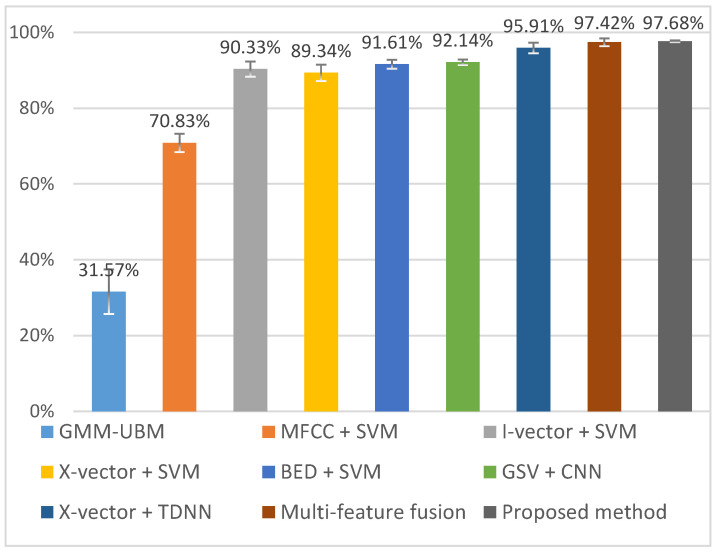
Comparison of the ACC with baseline methods.

**Figure 9:**
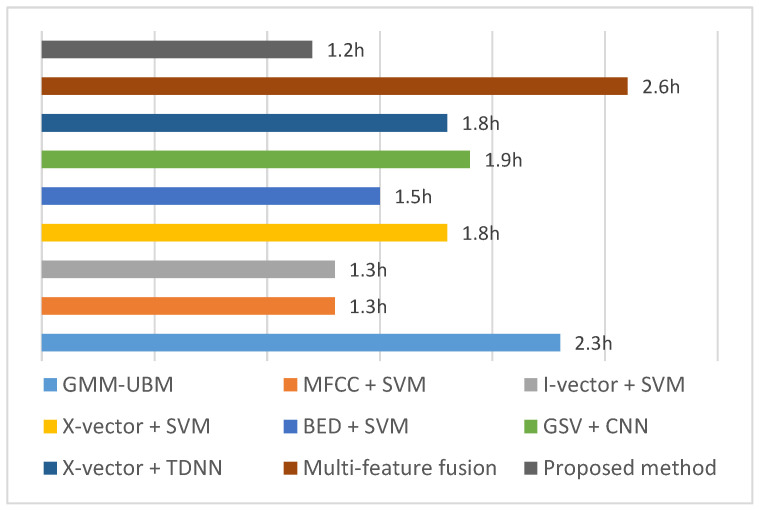
Comparison of the training time with baseline methods.

**Figure 10:**
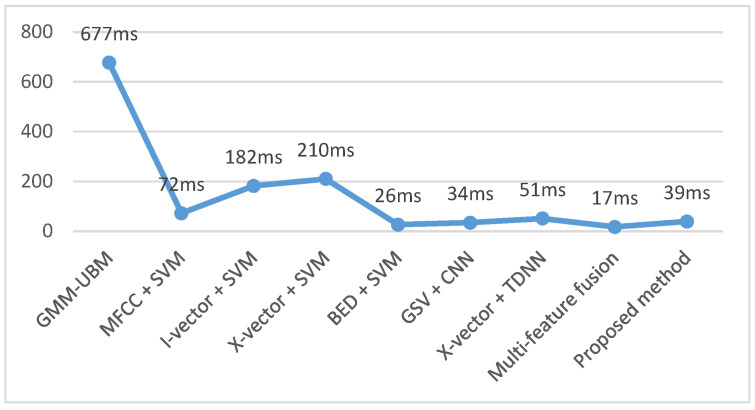
Comparison of the inference time with baseline methods.

For the accuracy comparison between this method and the baseline methods on the CCNU_Mobile dataset, Table 5 shows the mean values of the 10 results obtained from the experiments for each method. From the experimental results in Figure 8, it can be seen that the deep-learning-based methods generally outperform the traditional methods, and the choice of features can have an impact on recognition under the same model selection; however, the magnitude of the impact is not significant. As shown in Figure 8, our proposed method obtains the highest ACC while demonstrating smaller variance in multiple repetitions of the experiment relative to other deep learning methods, which implies that our proposed method achieves more stable recognition performance. In the classification of 45 categories of recording devices, the proposed method achieves a recognition rate of 97.68%, corresponding to approximately 5626 correctly identified samples among 5760 test audio samples. As shown in Figure 9 and Figure 10, although the performance of our method is not optimal in terms of inference time, it remains in the same order of magnitude as the methods with the shortest inference time, while the training time is significantly reduced.

#### 5.5.2. Ablation Experiments of Attention Mechanisms

In order to verify the effectiveness of the three attention mechanisms, four experiments were conducted, in which three attention mechanisms were present and one of them was removed. It can be seen from the experimental results in Table 6 that when one of the three attention mechanisms is absent, the model without a spatial attention mechanism obtains the worst effect, with an accuracy rate reduced by 0.2% compared with the model without a modal attention mechanism and the model without a temporal attention mechanism. This result shows that the spatial attention mechanism has the greatest influence on the network model investigated in this paper and that the spatial attention mechanism is also the model that requires the most network parameters. When the three attention mechanisms exist simultaneously, the model achieves the best effect, reaching 97.6%, directly proving the effectiveness of the three attention mechanisms proposed in this paper and indirectly proving that the three attention mechanisms achieved their respective intended effects.

#### 5.5.3. Validation Experiments of the RD-TCN Temporal Feature Extraction Network

In order to verify the effectiveness of the RD-TCN network, comparison experiments were conducted.The parameters of the ordinary TCN-based temporal feature extraction and the RD-TCN-based temporal feature extraction are shown in Table 4. The spatial feature extraction network adopts a structure of a CNN with the same network parameters as in Table 4.

In the RD-TCN temporal feature extraction network, the original residual blocks of the TCN network are replaced with residual dense blocks. From the experimental results in Table 7, it can be seen that the recognition accuracy of the ordinary TCN-based temporal feature extraction network reaches 97.4%, while the TCN network with the addition of residual dense blocks reaches 97.6%, which is an improvement of 0.2% compared with the ordinary TCN temporal feature extraction network, proving that the RD-TCN temporal feature extraction network is effective.

#### 5.5.4. Experiments for the Joint Loss Function

To optimize the model training process, we used the strategy of joint loss function, which was optimized by combining three losses. To explore the effectiveness of the joint loss function, we compared it with a model using a single cross-entropy loss function and tested the optimal parameters by controlling the proportional coefficients.

Table 8 shows the experimental results for the loss function settings. The ACC for all experiments is higher than 97.1% when using the joint loss function optimization strategy. The joint loss function optimization strategy significantly outperforms the network model using a single loss. The experimental results show that the network can converge better by co-optimizing multiple losses. Experiments comparing the joint loss function with different proportional coefficients shows that the network model can achieve the best results when the proportional coefficients are (0.25, 0.5, 0.25) and (0.25, 0.25, 0.5), reaching 97.6%, which indicates that the proper allocation of proportional loss coefficients can achieve better results.

## 6. Conclusions

In this paper, we propose a spatiotemporal representation learning method with a multi-attention mechanism for recording device source identification. In this paper, we used multiple features as input, which is a kind of multiple knowledge representation; this strategy effectively improves the recognition accuracy and contributes to the generalization ability and interpretability of the model [37]. The two features used were extracted from different abstraction levels, among which GSV is extracted based on MFCC, which is an information supplement based on a priori domain knowledge and eliminates category-independent information in the extraction process to avoid the interference of speech content or speaker information, and enriches the feature information in terms of probability density distribution information. In terms of structure, the method includes temporal and spatial feature reconstruction, temporal and spatial feature extraction, temporal and spatial feature fusion, and joint loss calculation stages. In terms of the contributions of this paper, first, feature reconstruction of temporal and spatial features was performed using temporal attention and spatial attention, respectively, significantly highlighting the temporal and spatial information of device source features and subsequently optimizing the fusion of both features using the branch attention mechanism. Secondly, in order to solve the problem of long training time required for existing deep learning models for device identification, in this paper, we used an RD-TCN network for temporal feature extraction, which also improved the recognition speed of the model on the basis of improving the accuracy rate.

In terms of experimental performance, the present method shows a small improvement in recognition accuracy relative to methods proposed in our previous work; however, the training time of this method is significantly reduced, and its performance is more stable. The recognition performance on this dataset is close to saturation, and even small improvements are meaningful, so we will subsequently explore application-oriented and effective recognition methods. In future work, we will further optimize the model to obtain more significant recognition performance improvement. However, the present method still has some shortcomings in the feature extraction stage, which require the early extraction of two features before model training, increasing the complexity of the application. We will investigate how to extract more expressive features and research feature extraction methods that are better-matched to the task of recording device identification. In terms of identification models, we will explore the application of self-supervised learning in this area and attempt to improve the application of transformer-based representational learning in recording device identification. We will also improve our method to be applicable to more complex recognition scenarios to promote additional applications.

## Figures and Tables

**Table 1:** A summary of notations.

Notation	Description
A	Audio signal
fl	Frame length
fs	Frame shift
X $=\left\{x_{1},x_{2},\ldots ,x_{T}\right\},x_{t}\in R^{M}$	MFCC features extracted after framing of samples
*K*	Number of Gaussian components
G $\in R^{M\times K}$	GSV features of samples
Conv	Convolution layer
MP	Maximum pooling layer
FC	Fully connected layer
σ	Sigmoid activation function
δ	ReLU activation function
w,W	Weights
λ={w,μ,Σ}	Model parameters of GMM
$L_{total},L_{spa},L_{tem},L_{cla}$	Components of the loss function
α,β,γ	Proportion coefficients of the three losses

**Table 2:** Brands and models of recording device in the CCNU_Mobile dataset.

Brand	Models
APPLE	iPhone6(4), iPhone6s(3), iPhone7p, iPhoneX, iPhoneSE, iPad7, Air1, Air2(2)
XIAOMI	mi2s, note3, mi5, mi8, mi8se(2), mix2, redmiNote4x, redmi3S
HUAWEI	Nova, Nova2s, Nova3e, P10, P20, TAG-AL00
HONOR	honor7x, honor8(3), honorV8, honor9, honor10
VIVO	x3f, x7, y11t
ZTE	C880a, G719c
SAMSUNG	S8, sphd710
OPPO	R9s
NUBIA	Z11

**Table 3:** Network parameters of the three attention mechanisms.

Spatial Attention Mechanism	Temporal Attention Mechanism	Branch Attention Mechanism
Conv(16, (3*3), (2*2))	Conv (16, (5*5), (3*3))	Conv (16, (3*3), (1*2))
Conv(32, (3*3), (2*2))	Conv (32, (3*3), (3*3))	Conv (32, (5*5), (1*3))
Pooling ((3*3), (2*2))	Pooling ((5*1), (5*1))	Pooling ((1*5), (1*5))
Flatten	Pooling ((7*1), (7*1))	Pooling ((1*7), (1*7))
Dense (2496)	Flatten	Flatten
Reshape (39*64)	Dense (650)	Dense (2)
Multiply	Reshape (650*1)	Reshape (2*1)
/	Multiply	Multiply

**Table 4:** Network parameters of the deep feature extraction networks.

Spatial Feature Extraction	Temporal Feature Extraction Network	
CNN	TCN Block	RD-TCN Block
Conv (6, (5*5), (1*1))	Conv (6, 5, 1)	Conv (6, 5, 1)
Pooling ((2*2), (2*2))	Conv (6, 5, 1)	Conv (6, 5, 1)
Conv (16, (5*5), (1*1))	add	Conv (6, 5, 1)
Pooling ((2*2), (2*2))	Conv (16, 5, 1)	add
Conv (40, (5*5), (1*1))	Conv (16, 5, 1)	Conv (16, 5, 1)
Pooling ((2*2), (2*2))	add	Conv (16, 5, 1)
Flatten	Conv (40, 3, 1)	Conv (16, 5, 1)
FC (1024)	Conv (40, 3, 1)	add
/	add	Conv (40, 3, 1)
/	/	Conv (40, 3, 1)
/	/	Conv (40, 3, 1)
/	/	add

**Table 5:** Comparison of identification accuracy with baseline methods.

Method	ACC	Training Time	Inference Time
GMM − UBM	31.57 ± 11.65%	2.3 h	677 ms
MFCC + SVM	70.83 ± 4.88%	1.3 h	72 ms
I-vector + SVM	90.33 ± 3.93%	1.3 h	182 ms
X-vector + SVM	89.34 ± 4.26%	1.8 h	210 ms
BED + SVM	91.61 ± 2.41%	1.5 h	26 ms
GSV + CNN	92.14 ± 1.40%	1.9 h	34 ms
X-vector + TDNN	95.91 ± 2.76%	1.8 h	51 ms
Multifeature fusion	97.42 ± 2.05%	2.6 h	**17 ms**
**Proposed method**	**97.68 ± 0.47%**	**1.2 h**	39 ms

**Table 6:** Comparison of the attention mechanism selection.

Model	ACC
Without temporal attention mechanism	97.2%
Without spatial attention mechanism	97.0%
Without branch attention mechanism	97.2%
**Model with three attention mechanisms**	**97.6%**

**Table 7:** Comparison experiment between RD-TCN and ordinary TCN.

Model	ACC
Model with ordinary TCN block	97.4%
**Model with RD-TCN block**	**97.6%**

**Table 8:** Experiments for the loss function setting.

Loss Function Setting	Proportional Coefficients (α,β,γ)	ACC
Single loss function	0, 0, 1	96.7%
Joint loss function	0.5, 0.25, 0.25	97.1%
**Joint loss function**	**0.25, 0.5, 0.25**	**97.6%**
**Joint loss function**	**0.25, 0.25, 0.5**	**97.6%**
Joint loss function	0.4, 0.2, 0.4	97.3%
Joint loss function	0.2, 0.6, 0.2	97.4%

## Data Availability

Data will be made available upon reasonable request.

## Abbreviations

The following abbreviations are used in this manuscript:

MFCC	Mel frequency cepstrum coefficient
BED	Band energy difference
VAD	Voice activity detection
SVM	Support vector machine
GMM	Gaussian mixture model
DNN	Deep neural network
GSV	Gaussian super vector
VQ	Vector quantization
SRC	Sparse representation-based classifier
2D	Two-dimensional
FFT	Fast Fourier transform
DCT	Discrete cosine transform
EM	Expectation maximization
RNN	Recurrent neural network
